# Reducing Bias in Auditory Duration Reproduction by Integrating the Reproduced Signal

**DOI:** 10.1371/journal.pone.0062065

**Published:** 2013-04-16

**Authors:** Zhuanghua Shi, Stephanie Ganzenmüller, Hermann J. Müller

**Affiliations:** 1 General and Experimental Psychology, Department Psychology, LMU Munich, Munich, Germany; 2 Graduate School of Systemic Neuroscience, LMU Munich, Munich, Germany; 3 Department of Psychological Science, Birkbeck College (University of London), London, United Kingdom; Duke University, United States of America

## Abstract

Duration estimation is known to be far from veridical and to differ for sensory estimates and motor reproduction. To investigate how these differential estimates are integrated for estimating or reproducing a duration and to examine sensorimotor biases in duration comparison and reproduction tasks, we compared estimation biases and variances among three different duration estimation tasks: perceptual comparison, motor reproduction, and auditory reproduction (i.e. a combined perceptual-motor task). We found consistent overestimation in both motor and perceptual-motor auditory reproduction tasks, and the least overestimation in the comparison task. More interestingly, compared to pure motor reproduction, the overestimation bias was reduced in the auditory reproduction task, due to the additional reproduced auditory signal. We further manipulated the signal-to-noise ratio (SNR) in the feedback/comparison tones to examine the changes in estimation biases and variances. Considering perceptual and motor biases as two independent components, we applied the reliability-based model, which successfully predicted the biases in auditory reproduction. Our findings thus provide behavioral evidence of how the brain combines motor and perceptual information together to reduce duration estimation biases and improve estimation reliability.

## Introduction

For everyday actions, we must be able to incorporate multiple sensory feedbacks for fine-tuned movement in space and time. Precise timing, especially in the sub-second range, is crucial for everyday activities like walking, speaking, or playing sports and making music [1]. However, research has revealed that our perception of time can be distorted in various ways, such as by a voluntary action [2], [3], the emotional state of the observer [4], [5], or repeated presentation [6]. Also, perceived durations in different modalities can vary substantially. For example, an auditory interval is often judged or produced longer than a visual interval with the same length [7]–[11]. Timing for action can also be different from timing for perception [12]. For instance, motor reproduction of an auditory duration relying only on kinesthetic information has been reported to be overestimated by about 12% [8], which is larger than the biases found in traditional perceptual comparison tasks. Moreover, not only the perceived time of an on-going action, but also the perceived time of an event that immediately follows an action can be distorted by the action. For example, the first second immediately after a saccadic or an arm movement is often perceived as longer than subsequent seconds, which is known as the chronostasis illusion [3], [13], [14]. Distortions induced by actions have also been shown in the opposite direction, such as compression of time during saccadic movements [2], [15].

Given that perceived time is far from veridical and time estimation can be easily biased by various factors, our brain encounters challenges to integrate different sources of temporal information so as to enable accurate timing for multisensory or sensorimotor events. When inter-sensory biases are detectable (e.g., a longer auditory signal than a visual signal in an echo environment), it has been consistently found that the sensory system may recalibrate itself to maintain internal consistency (for a recent review, see [16]). How the sensory system recalibrates itself is still controversial. Some groups have proposed that the discrepancy in sensory estimates is recalibrated proportional to their reliabilities [17]–[19]. Based on developmental studies, on the other hand, Gori and colleagues [20] have argued that the recalibration depends on the robustness, rather than the reliability, of the senses. Other researchers have also proposed alternative accounts, for instance, that the calibration is based on prior knowledge about the probability of the signals being biased [16], [21], or on fixed-ratio adaptation, whereby cues adapt toward one another at a fixed ratio regardless of cue reliability [22]. Rather than recalibrating the sensory input, the brain could also decide to primarily rely on one sense and ignore information from other senses, as suggested earlier by the modality dominance hypothesis [23]. Relying only on the estimate from one reliable modality could shield from noises and biases from unreliable or inaccurate senses. Note that recalibration or modality dominance in multimodal processing is needed mainly for maintaining an internal, consistent representation [16]. However, recalibration does not solve the bias problem because biases are inherited from individual sensory estimates. Thus, the system still faces the problem of having to reduce the bias. This is particularly true for large differences and biases in perceptual and motor estimates of the same time interval.

When estimation biases do not cause internal discrepancy, the question of how the brain deals with multiple temporal estimates is still poorly understood. In the spatial domain, reliability-based optimal integration models, such as Maximum Likelihood Estimation (MLE), have successfully predicted the effects of multimodal integration for various situations, including visual-haptic size estimation, audio-visual localization, etc. (for a recent review, see [24]). The optimal integration model assumes that our sensory system combines multiple unbiased estimates in a linear weighted fashion, with each weight set in proportion to the reliability of the corresponding sensory source. The integration is optimal since the weighted combination minimizes the estimation uncertainty, that is, maximizes the estimation reliability. However, with regard to the multimodal temporal domain, the findings are rather mixed. A study using temporal-order judgments (TOJ) has found that the MLE model could account well for performance in a bimodal, audio-tactile TOJ task [25]. However, using a temporal-bisection task, Burr, and colleagues [26] found that the MLE model only fitted roughly with their observed result pattern. Employing an apparent motion paradigm and an implicit measure of perceived time interval, Shi and colleagues [9] found that while audio-visual intervals were integrated in an optimal manner, the predicted reduction of the variability of the estimates in the audio-visual condition was not observed. A pattern of well predicted temporal estimates, but missing reductions of variability has also been confirmed by other studies using a temporal bisection task [27] or a visual-tactile reproduction task [28]. Thus, compared to spatial multimodal integration [29]-[31], the predictions of the reliability-based model are less consistent and inconclusive with regard to multimodal temporal integration. In particular, there is a lack of investigation of sensorimotor temporal integration.

Given this, the present study was designed to test the reliability-based cue integration model for sensorimotor temporal integration, in particular for auditory reproduction. According to the reliability-based MLE model, the estimate of the auditory reproduction (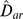) for a given standard auditory duration (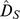) results from a linear weighted combination of the perceptual comparison (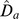) and pure motor reproduction (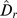). Assuming that the perceptual and motor estimates are statistically independent of each other, the MLE estimate of the auditory reproduction is given as follows:
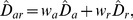
(1)

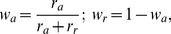
(2)where 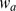 and 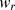 are the correspondent weights and 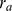 and 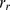 are the reliabilities of the estimates, where reliability is defined as the inverse of its respective variance, 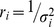. With these weights the variance of the auditory reproduction 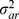 is given by 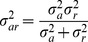
(3)


The variance is the minimum possible for any linear combination and is lower than the variances of the pure perceptual and motor estimates, 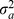 and 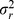. In other words, the reliability of the MLE estimate is the maximum. Note that minimizing variability (i.e., maximizing reliability) of the auditory reproduction does not guarantee reduction of the bias. Rather, derived from Eq. (1) and (2), the auditory reproduction bias, 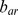, becomes a weighted average of the perceptual bias 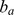 and motor bias 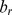:
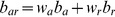
(4)


If the system does not know where biases come from and if biases vary randomly around the true value, a linear weighted combination may, in general, reduce the bias, even though the combined sensorimotor estimate is not optimal in terms of accuracy.

Testing whether the sensory system uses a reliability-based integration to minimize variability and reduce biases in the auditory duration reproduction, we must compare the goodness of the predictions among the MLE, the auditory dominance, and the motor dominance models in the following aspects: (1) the predicted variances should be close to the observed variances; (2) the predicted estimates should be highly correlated with the observed estimates; (3) for an ideal prediction, the predicted estimates should be equal to the observed estimates. In other words, the slope of a linear regression (without an intercept) between the predicted and observed estimates should be close to 1; (4) the predicted errors measured by root mean square errors (RMSEs) should be smallest.

**Figure 1:**
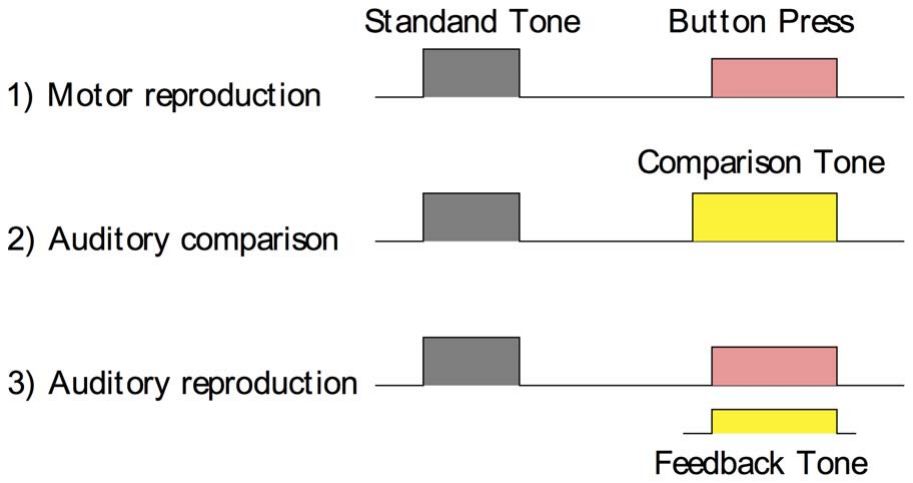
Schematic illustration of three estimation tasks, which all started with the presentation of an auditory standard duration. In the motor reproduction and auditory reproduction tasks, participants had to reproduce the standard duration by pressing a button. In the auditory reproduction task, the reproduced tone was synchronous with the button press. In the comparison task, an auditory comparison stimulus was presented and participants had to indicate which tone was perceived as longer.

Thus, we conducted two experiments and compared duration biases and variances among three different tasks: motor reproduction, auditory duration comparison, and auditory reproduction (Figure 1).

The auditory comparison and motor reproduction tasks aimed to measure biases and variances for perceptual and motor timing, respectively. In the auditory comparison task, participants were presented with two tones and had to indicate which one was longer. In the motor reproduction task, participants had to press a button as long as the duration of the (previously presented) standard auditory tone. The third, auditory reproduction task was a bimodal (i.e., perceptual and motor) condition: participants had to press a button to produce a tone of the same duration as the previously presented auditory standard. Note that in both reproduction tasks, there is kinesthetic and tactile (touch sense) feedback during the button press. A previous sensorimotor tapping study [32] has shown that blocking the peripheral feedback leads to an increase of the variability in synchronizing the pacing signal with the tap. Here, however, we consider motor action and peripheral touch feedback as one, unitary motor component. This does not compromise our aim of examining how reproduced auditory feedback influences time estimation. In Experiment 1, we compared estimations among the three tasks (duration comparison, pure motor reproduction, and auditory reproduction) for a single auditory standard duration (1 second). To vary the reliability of the signals, in Experiment 2, we manipulated comparison/reproduced tone signal-to-noise ratio (SNR) block-wise. In addition, we mixed two standard durations, 800 and 1200 ms, together to increase task uncertainty.

## Results

### Experiment 1

**Figure 2:**
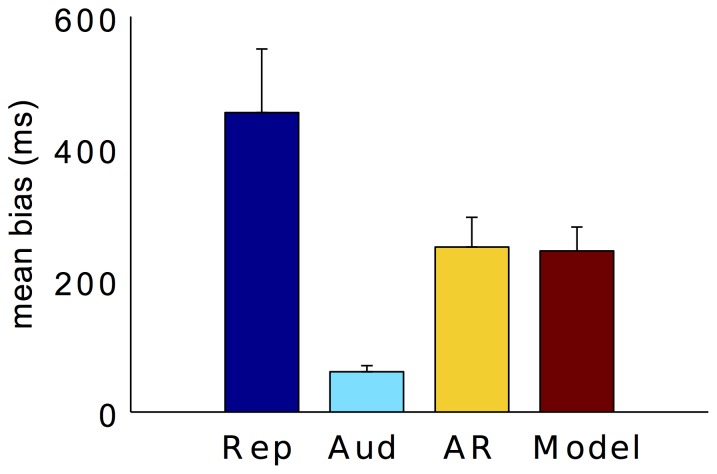
Mean biases (with±1 standard errors) for the pure motor reproduction (blue bar), auditory comparison (cyan bar), auditory reproduction (yellow bar), and predicted according to the MLE model (red bar) in Experiment 1.

Figure 2 depicts the mean biases for three tasks (pure motor reproduction, auditory comparison, and auditory reproduction). Pure motor reproduction produced the largest overestimation (454±96.9 ms). For the auditory comparison task, on the other hand, the bias (60±9.5 ms) was the smallest, though it was still significantly greater than zero, *t*(10) = 6.69, *p*<0.01. The overestimation in the comparison task was probably due to the low intensity of the comparison signal (46 dB) compared to the standard signal (68 dB), as shown previously [33]. Bonferroni corrected pairwise comparison revealed a significant difference between motor reproduction and auditory comparison (*p*<0.01), as well as one between auditory comparison and auditory reproduction (*p*<0.01). There was also a marginally significant difference between pure reproduction and auditory reproduction (*p* = 0.052). Based on Equations (2) and (4), we then calculated the predicted mean bias of auditory reproduction according to the MLE model. The predicted bias did not differ from the observed bias (*p* = 0.88).

**Figure 3:**
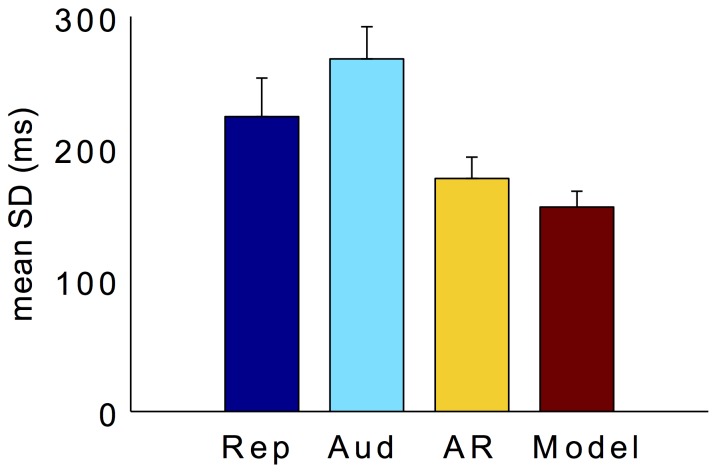
Mean SDs (with±1 standard errors) for the pure reproduction (blue bar), auditory comparison (cyan bar), auditory reproduction (yellow bar), and predicted according to the MLE model (red bar) in Experiment 1.

However, the pattern is different when looking at the estimation variability indicated by the standard deviations (SDs) (Figure 3). The mean SDs differed significantly among the three tasks, as confirmed by a repeated measures ANOVA, *F*(1.33,13.33) = 219.33, *p*<0.05 (Greenhouse-Geisser corrected). Bonferroni corrected pairwise comparisons revealed the variance to be significantly smaller in the auditory reproduction than in the auditory comparison task (*p*<0.05). More interestingly, the predicted mean variance according to the MLE model did not differ from the observed mean variance of the auditory reproduction (*p* = 0.09).

We further compared the goodness of fit for three different models (MLE, auditory/motor dominance) using three additional measures: the slope of the linear regression (without an intercept) between the observed and predicted biases, the correlation between the predicted and observed biases, and the mean predicted error RMSE. Results are shown in Table 1. Both the MLE and the motor dominance model show a high correlation between the predicted and observed biases. However, only for the MLE model the slope was close to 1. In addition, RMSE was the smallest in the MLE model. Clearly, the prediction of the MLE model is better than that of the two dominance models.

**Table 1:** Goodness of predictions based on the slope (±95% confidence interval), correlation coefficient r (*p<0.05), and RMSE for the MLE, motor dominance, and auditory dominance models in Experiment 1.

Models	Slope±95% CI	r	RMSE
MLE	0.98±0.29	0.62 *	110
Motor dominance	0.48±0.16	0.66 *	305
Auditory dominance	3.14±2.01	−0.26	239

### Experiment 2

**Figure 4:**
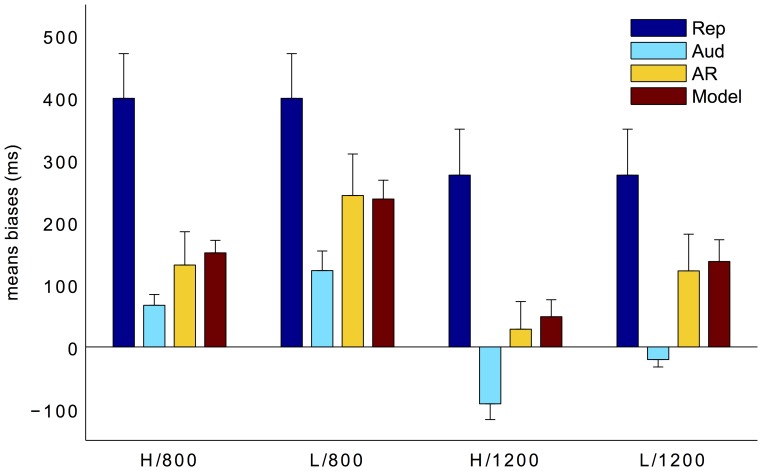
Mean biases (with±1 standard errors) for pure reproduction (blue bars), auditory comparison (cyan bars), auditory reproduction (yellow bars), and predicted according to the MLE model (red bars), as a function of the SNR and standard duration in Experiment 2. H and L denote the high and low SNR conditions, 800 and 1200 the short and long standard durations.

In Experiment 2, we increased task uncertainty by introducing two standard intervals (i.e., 800 and 1200 ms) and two signal-noise ratios (SNRs) in the compared/reproduced tones (High-SNR: 11 dB, Low-SNR: −14 dB). Figure 4 depicts the mean biases for Experiment 2. A three-way repeated measures ANOVA with length of duration, SNR, and task as factors revealed that the bias was significant influenced by all three factors: the length of duration, *F*(1, 9) = 24.08, *p*<0.01; SNR, *F*(1,9) = 23.31, *p*<0.01; and task, *F*(2,18) = 15.43, *p*<0.01. The low SNR increased the positive bias in the duration estimation. The higher overestimation for the short duration (800 ms) than for the long duration (1200 ms) confirmed previously reported range and regression effects [34]–[37], which suggests that participants tend to be biased towards the center of the stimulus range. In our case, due to the random mixing of the short and long standard duration trials, estimation of the short duration was biased towards the long duration and vice versa. Further post-hoc Bonferroni multiple-comparison tests indicated that the biases differed significantly among the three tasks (all p<0.05), with the lowest bias in the comparison task and the highest in the motor reproduction task. There was also one (and only one) significant interaction between SNR and task, *F*(2,18) = 10.49, *p*<0.01. This was mainly due to the fact that there was no auditory and noise signals in the pure motor reproduction. Most interestingly, the predicted biases according to the MLE model did not differ from observed auditory reproduction biases (all p>0.1, Figure 4).

Similar to Experiment 1, we compared the goodness of fit for the three possible models (MLE, auditory/motor dominance) with three different measures. We pooled all data (conditions) together to make a strict test. Results are shown in Table 2. No correlation between the observed and predicted biases for the auditory dominance model clearly indicates its bad prediction. On the other hand, the correlation was highest in the motor dominance model, yet its regression slope was only half (0.47) and RMSE was the largest one. Taking three indicators together, the MLE model best predicted the data, which corroborated the finding in Experiment 1.

**Table 2:** Goodness of predictions based on the slope (±95% confidence interval), correlation coefficient r (*p<0.05), and RMSE for the MLE, motor dominance, and auditory dominance models in Experiment 2.

Models	Slope±95% CI	r	RMSE
MLE	1.01±0.24	0.70 *	129
Motor dominance	0.47±0.09	0.81 *	242
Auditory dominance	0.57±0.65	0.21	217

**Figure 5:**
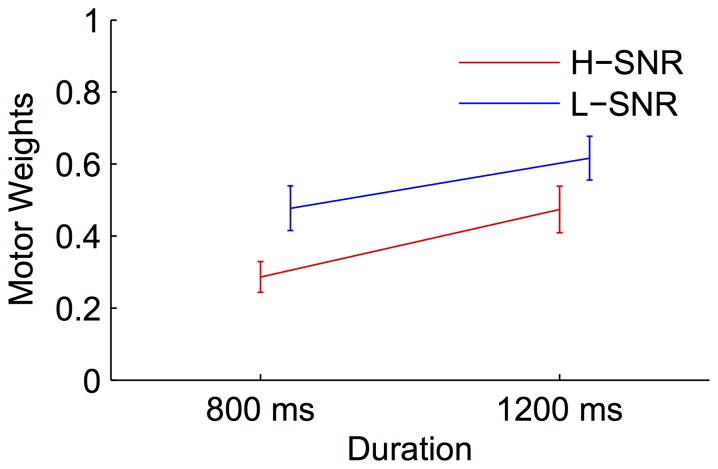
Mean predicted motor weights as a function of the duration length and SNR for the auditory reproduction task. H-SNR and L-SNR denote the high and low SNR conditions, respectively.

Further, we estimated weights for the different conditions. Figure 5 illustrates the systematic changes of motor weights with duration length and SNR. A repeated measures ANOVA revealed that both SNR and duration significantly influenced the weight adjustments, with greater reliance on motor timing for the long compared with the short duration, *F*(1,9) = 22.17, *p*<0.01, and higher weights on motor timing for the low SNR (−14 dB) than for the high SNR (11 dB) condition, *F*(1,9) = 24.95, *p*<0.01. This is because the long duration and, respectively, the low SNR auditory feedback exhibited larger variability than the short duration and, respectively, the high SNR auditory feedback. There was no interaction between the two factors, *F*(1,9) = 1.2, *p* = 0.4.

**Figure 6:**
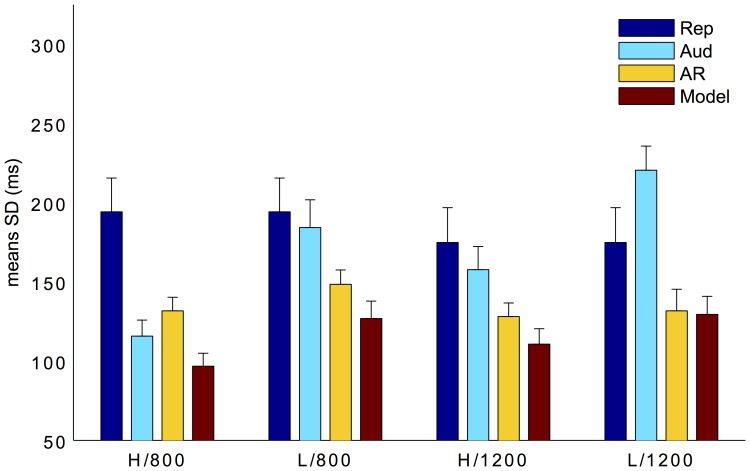
Mean SDs (with±1 standard errors) for pure reproduction (blue bars), auditory comparison (cyan bars), auditory reproduction (yellow bars), and predicted according to the MLE model (red bars), as a function of standard duration and SNR in Experiment 2. H and L denote high and low SNRs, 800 and 1200 short and long standard durations.

The pattern of variances indicated by the SDs is depicted in Figure 6, which shows that SDs are lower in the high compared to the low SNR conditions, and in the auditory reproduction compared to the pure motor reproduction condition. This pattern was confirmed by a three-way repeated measures ANOVA, which revealed significant effects for SNR, *F*(1,9) = 21.94, *p*<0.01, and task, *F*(2,18) = 5.42, *p*<0.05, but not for length of the standard duration, *F*(1,9) = 0.15, *p* = 0.7. Post-hoc Bonferroni tests indicated that the mean SD was lowest in the auditory reproduction task (all p<0.05). As in Experiment 1, we compared predicted variability based on the MLE model with observed variability, as additional confirmation criterion for reliability based integration. The observed variability and predicted variability did not differ for the long standard durations (both p>0.1), being in agreement with reliability based model predictions. However, for the short durations, there were significant differences between predicted and observed variability for high SNR, *t*(9) = 5.70; *p*<0.05, and for low SNR, *t*(9) = 3.09, *p*<0.05. This suggests that the integration was suboptimal for the short durations.

## Discussion

We examined how the brain incorporates different sources of timing information in duration estimation. We compared estimation biases in an auditory comparison, motor reproduction, and auditory reproduction task. We found two major results: First, while perceptual comparison of two auditory tones was fairly accurate, reproduction of an auditory tone yielded consistent overestimation. The overestimation was reduced when the reproduction produced a tone feedback, though even then it remained larger compared to the auditory comparison task. Second, we fitted the results with the MLE optimal integration model, which yielded a good prediction for the estimation bias.

Our finding of a large difference between perceptual comparison and motor reproduction for the same physical duration clearly favors distributed timing mechanisms [38]–[42]. It is well established that perceived duration in different modalities can vary, such that sounds are perceived as longer than lights or tactile vibrations of equal physical duration [7]–[9], [43]–[45]. Some other recent studies have also pointed to different mechanisms for motor and sensory timing. For example, differences between perceptual and motor timing have been demonstrated by a delay manipulation prior to the task [12], [46]. Also, an opposite temporal distortion pattern between perceptual and motor time estimations has been reported for novel versus repeated stimuli [47]. It should be mentioned that many other studies favor a common mechanism for motor and sensory timing [48]–[50]. Most of these studies, however, used slightly different tasks (e.g., rhythmic tasks) and often gap intervals. Unlike reproduction with a continuous button press, the perceptual task (defined by two brief stimuli) and the motor task (demarcated by two taps) with gap intervals are more “similar” to each other, as the gap information is likely modality-independent and processed by a common mechanism. In contrast to these paradigms, we used filled intervals for both perceptual comparison and motor reproduction. From this aspect, our findings of a strong difference between the motor reproduction and auditory comparison tasks argue in favor of a perception-action dichotomy in the timing mechanisms involved.

The strong over-reproduction in the motor task (about 38%) seems striking. Walker and Scott [8], some time ago, reported an overestimation of auditory durations by about 12%, though they did not provide any explanation for this finding. It has been suggested that motor reproduction might include an additional component of motor planning in time encoding [51]. Temporal reproduction has been thought to consist of two consecutive processes: waiting until the elapsed time is “close enough” to the standard, at which point a response is initiated, and then executing the response (i.e., button press), which again takes time (see also Wing and Kristofferson's model [52]). However, such an explanation cannot account for our finding of a pronounced overestimation, since the initiation and termination of a response in our *filled*-reproduction task could be both delayed and the delays might cancel each other. Even without any cancelation, the large over-reproduction is unlikely due to the motor planning time. However, the additional noise generated by the motor control and planning processes is most likely present, as indicated by the estimation variances.

Both estimation biases and variances were decreased in the auditory reproduction compared to the motor reproduction task. The reproduced auditory signal seems to contribute to the final reproduction by reducing the bias and variability. Using the reliability-based MLE model, we found that the quantitative model successfully predicted the auditory reproduction biases, and it performed far better than either a motor dominance or a perceptual dominance model.

It should be noted that most studies using MLE or a more general Bayesian approach employed physical measures as their integration cues for multimodal integration. Some small external discrepancies were often introduced during the experiments. The implicit assumption of optimal integration, using external physical measures, is that all sensory estimates are unbiased. Disregarding biases allows one to focus on minimizing variance as an optimality criterion [53]. As reviewed earlier, subjective and physical durations have been shown to be quite different and temporal biases are ubiquitous. If the quantitative model had considered only physical durations, it would not have provided any useful predictions in our case, because the physical durations were identical. In the present study, we explicitly modeled biases (see Equation 4). By integrating two (or more) estimates, the system can reduce the variability of the final estimate. This idea goes along with the recent memory-mixing account [54], which suggests that our brain might combine multiple signal durations together for time estimation. However, integrating or mixing multiple biased estimates may reduce the accuracy of the final estimate. For example, in our study, the bias in auditory reproduction was larger than that in the pure auditory comparison. Thus, the estimation would have been better in terms of accuracy if the system only trusted the auditory comparison. In this sense, the linear weighted integration is not optimal if estimates have biases. Of course, without any external feedback, the system does not know if the sensory or/and the motor estimate is biased. Using a weighted averaging method in this situation may reduce the variability of the estimate, though it may not lead to the best-unbiased estimate.

Integration of subjective estimates has also been tested recently with visual and tactile duration judgments [28], for which the bimodal duration was predicted successfully by the MLE model. However, the variability of the bimodal condition was far from “optimal”, not showing the theoretically predicted improvement. Interestingly, several recent studies of multimodal temporal integration [9], [26]–[28] confirmed that the MLE prediction of the bimodal variability was suboptimal: in general, the predicted variance was smaller than the observed one. This was also the case in our Experiment 2, in which the predicted variances for the short standard durations were significant lower than the observed variances. The reason for this suboptimal integration is not clear at present. It has been suggested that the assumption of Gaussian noise might not be appropriate for timing tasks [26]. Alternatively, variability in the auditory reproduction task may not be further reduced for the short standard durations, due to the accuracy limits of the motor system. It is also possible that time estimates from different sensory (motor) modalities are not completely distributed and statistically independent, as hinted at by the internal common time processing literature [49], [50], [55]–[58]. When sensory estimates are correlated, the optimal weights and reliability could dramatically deviate from independent optimal integration [59].

It should be mentioned, however, that the present study only investigated the integration of auditory reproduction. Several researchers have argued that there might be a privileged link between the auditory and the motor system in the time domain, allowing for a direct integration of auditory information into the motor system [39], [60], [61]. In an fMRI tapping study, for example, it has been shown that tapping to auditory stimuli is driven by a reliable internal movement rhythm. But during tapping to visual stimuli participants rather relied on an inefficient and computational demanding control network [61]. In a previous study, we have also found that while offset-delayed auditory feedback led to a decrease in duration reproduction, there was no effect of offset-delayed visual feedback [11]. Further, it has been shown that initiating an action during a temporal-bisection task could enhance auditory temporal sensitivity, while there was no effect of an action on visual temporal sensitivity [62]. Therefore, the integration of other-modality sensory feedback (visual or tactile) during duration reproduction might have different results, which is definitely intriguing for future studies.

In summary, the present study investigated subjective differences between perceptual and motor timing, and their integration mechanism. There was strong overestimation in the motor and auditory reproduction tasks. When a reproduced auditory signal was given during the reproduction, the overestimation bias was reduced, though it was still larger compared to the pure auditory comparison task. The reliability-based model successfully predicted the auditory reproduction bias for one and for multiple standard durations, as well as for the varying SNR conditions. The variability of the estimation was also reduced in the auditory reproduction task compared to the pure motor reproduction or perceptual comparison tasks. However, the observed variances did not reach the optimal level for the short duration conditions. To address this, the possibility of prior updates [34], [63] ought to be investigated in future studies to quantify sensorimotor time estimation more precisely.

## General Methods

### Subjects

21 naive volunteers (16 females, mean age 25.3 years) participated in the two experiments for payment (11 and 10 participants for Experiments 1 and 2, respectively). All participants had normal or corrected-to-normal vision; none of them reported any history of somatosensory disorders.

### Ethics Statement

All participants gave written informed consent in accordance with the Declaration of Helsinki (2008). Experiments were approved by the Ethics committee of the Psychology Department, LMU Munich.

### Stimuli and apparatus

All experiments were conducted in a dimly lit cabin (0.21 cd/m^2^). Auditory tones were the mainly used stimuli in the experiments. The standard tone was an 800 Hz, 68 dB tone presented for 1000 ms in Experiment 1; and an 800 Hz, 75 dB tone presented for 800 or 1200 ms in Experiment 2. The feedback and comparison tone was a 600 Hz, 46 dB tone in Experiment 1, and a 600 Hz, 74 dB and 49 dB tone for high and, respectively, low SNR conditions in Experiment 2. Additionally, pink noise was presented during the task (62 dB in Experiment 1 and 63 dB in Experiment 2). Thus the signal to noise ratio (SNR) of the comparison/feedback tone was 16 dB in Experiment 1, and 11 and −14 dB for the high- and, respectively, low-SNR comparison/feedback tones in Experiment 2. Stimulus presentation and data acquisition were controlled by a National Instrument PXI system, ensuring highly accurate timing (<1 ms). The experimental programs were developed using Matlab and Psychophysics Toolbox [64]. Tones and pink noise were delivered to participants via speakers imbedded in the monitor. The response button was placed on the table in-between the participant and the monitor. Reproduction times were measured using a response button, which participants pressed with their right-hand index finger. For the comparison task, left and right arrow keys were used for response acquisition.

### Procedure

In both experiments, we tested three different tasks: pure motor reproduction, duration comparison, and auditory reproduction (Figure 1).

In the duration comparison task, each trial started with a standard tone, defining a standard duration (1000 ms in Experiment 1, 800 or 1200 ms in Experiment 2). After a variable inter-stimulus interval randomly selected from 650–800 ms, a second comparison tone was presented. The duration of the comparison tone was randomly selected from seven preselected intervals, which were centered on the respective standard duration: they were selected systematically from around the standard duration, separated by steps of 10% of the Weber fraction. Thus, for the 1000-ms standard, comparison durations were 700, 800, …, 1300 ms; for the 800-ms standard, 560, 640, …, 1040 ms; and for the 1200-ms standard, 840, 960,…, 1560 ms. Participants were asked to compare the duration of the two tones and indicate whether they perceived the first or the second tone as longer, by pressing the left or right arrow key, respectively. In Experiment 2, two comparison tones differing in loudness were presented in block-wise manner.

In the duration reproduction tasks, again each trial started with a standard tone (the same as in the duration comparison task). Following the presentation of the standard tone, participants were asked to reproduce the duration as accurately as possible by button press, with reproduction duration demarcated by the onset and offset of the press action. In the auditory reproduction task, pressing the button produced a synchronous tone. In Experiment 2, two feedback tones differing in loudness were presented during the auditory reproduction task, manipulated in blocked-wise manner.

The three tasks were presented in separate blocks, with block order randomized across participants. In Experiment 1, there were 4 blocks of the comparison task, 2 blocks of the motor reproduction task, and 2 blocks of the auditory reproduction task. Each block consisted of 49 trials. In Experiment 2, blocks were split into two sessions run on separate days, due to the large number of to-be-completed trials. There were 2 [days] ×7 blocks of the comparison task, 2×2 blocks of the motor reproduction task, and 2×3 blocks of the auditory reproduction task. Each block consisted of 28 trials. Participants took a short break after every block. In addition, there was a short practice part introducing all three conditions, run prior to the formal experiment.

### Data analysis

For the duration comparison task, psychometric curves were fitted by cumulative Gaussian functions to each participant's responses. Points of subjective equality (PSEs) were then estimated from the 50% threshold points of the psychometric curves. The standard deviation (SD) was estimated from the cumulative Gaussian function [29], [30]. Note that the standard tone was always presented first; thus, the perceptual standard deviation would have to be adjusted by a constant multiplier 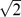 (see [65], [66]). However, since all three tasks started with the presentation of the standard tone (which participants would essentially memorize), this constant multiplier did not influence the model prediction. We therefore omitted it in the calculation. For the duration reproduction tasks, mean reproduced duration and standard deviation were calculated for each condition and individual participant. Extreme outliers, outside the upper 99% and lower 1% percentile, were removed from further analysis. The predicted biases and standard deviations were then calculated based on Equations (1) to (4).
